# Biomechanical assessment and comparison of fixation methods for bilateral sacroiliac joint luxation in 3D-printed feline pelvic bone models

**DOI:** 10.14202/vetworld.2024.1798-1802

**Published:** 2024-08-20

**Authors:** Tassanee Jaroensong, Kheira Lertjarugate, Natkamol Kumnuansil, Pimmada Puettimas, Pasinee Patanavibul, Suttipong Penpiratkul, Chaiyakorn Thitiyanaporn

**Affiliations:** 1 Department of Companion Animal Clinical Science, Faculty of Veterinary Medicine, Kasetsart University, Bangkok, 10900, Thailand; 2 Faculty of Veterinary Medicine, Kasetsart University, Bangkok, 10900, Thailand; 3 Orthopedic Surgery Unit, Kasetsart University Veterinary Teaching Hospital, Faculty of Veterinary Medicine, Kasetsart University, Bangkok, 10900, Thailand

**Keywords:** 3D-printed model, biomechanical testing, cats, sacroiliac joint luxation

## Abstract

**Background and Aim:**

Bilateral sacroiliac joint luxation, a condition primarily observed in cats, can significantly impact their quality of life. This study aimed to compare a control with three distinct fixation methods to identify the most robust fixation method capable of withstanding significant tensile stress.

**Materials and Methods:**

Twenty pelvic bone models of cats were made using a 3D printer with polylactic acid plastic. Each model was assembled by cutting the sacroiliac joints and pelvic girdle symphysis with a handsaw, then bonded with cyanoacrylate glue. 3D feline pelvic bone models were categorized into four significant groups, each consisting of five models. The study discovered that the three groups used distinct fixation methods: Two lag screws (DS), K-wires at the ilium wing and sacroiliac joints (TK), and K-wires at the sacroiliac joints (DK). The final group, not fixed, was the control.

**Results:**

The results were characterized further through a mechanical compression force test using a universal testing machine. The most robust method at the sacroiliac joints, the DK technique, sustained a maximum force of up to 183.86 N while maintaining the correct bone alignment. The fixation method is more accessible and faster to implement in comparison to the DS method.

**Conclusions:**

The DK group exhibited the greatest maximum load capacity among all groups. Sacroiliac joint luxation treatment can effectively be addressed using the K-wires fixation method. However, the DK need space of sacral body same as DS for fixation. Further clinical study should be performed.

## Introduction

In small animal clinics, pelvic injuries are frequently reported due to trauma [1–4]. Pain and pelvic narrowing might result in obstructed bowel movements, compromising the animal’s quality of life and even resulting in death. Sacroiliac joint luxation, a common pelvic injury, can occur on either side. Treatment options vary; conservative measures are employed when the ilium is slightly dislocated from the sacrum, whereas surgical intervention involving fixing the ilium to the sacrum is necessary under complete dislocation [5].

Fixing the ilium wing on the sacral wing’s facet with two lag screws is the standard method for stabilizing the sacroiliac joint [6–8]. Cats have a limited sacral area for secure screw insertion due to its decreased size (0.5 cm^2^) [6, 9]. Complications such as screw loosening and pelvic narrowing have been reported post-surgery [2, 10–12]. Several studies have examined sacroiliac joint injury fixation techniques in cats, but the biomechanics of these methods require further investigation [6, 7, 13–18]. Three-dimensional (3D)-printed models are frequently employed for fracture studies to minimize confounding factors compared to traditional dry or fresh bone methods [19–23]. 3D printing is used to create a physical model from computed tomography (CT) scan data. The screws and K-wires are employed for securing the sacroiliac joint.

This study aimed to assess the effectiveness of varying methods for repairing bilateral luxation of the sacroiliac joint in a feline pelvic model. Double screws, one transiliac pin, and two pins were used for the sacroiliac fixation.

## Materials and Methods

### Ethical approval

This study was conducted with 3D printed PLA feline pelvic models fixed with Kirschner wire and screws. The mechanical test was performed by a universal mechanical testing machine. Animals were not used in this study so, ethical approval of animal use was not necessary.

### Study period and location

This study was conducted 4 months, from March to June 2023, at the Faculty of Veterinary Medicine and Faculty of Engineering, Kasetsart University, Bangkok, Thailand.

### Model preparation

The pelvic model of a 4-kg cat was scanned using CT for reasons unrelated to this research. Digital imaging and communications in medicine files were converted to stereolithography files using the 3D Slicer 4.11.20210226 software (https://www.slicer.org). Twenty pelvic models were printed in 3D using polylactic acid (PLA) plastic material on a 3D printer (Ultimaker S5, Utrecht, Netherlands). The junction between the sacrum and ilium was cut using a saw blade. The pelvic models were then categorized into four groups: Control, double lag screws (DS), transilial pinning with one transiliosacral pinning technique (TK), and double K-wire transiliosacral pinning technique (DK).

### Fixation methods

In the control group, the sacroiliac joints of both models were joined using cyanoacrylate glue. In the DS group, both sacral body sites were drilled using a 1.5-mm drill bit, and the holes were tapped using a 2.0-mm tapper. Both sites of the ilium body were drilled with a 2.0-mm drill bit. A 2.0-mm screw was inserted from the hole in the ilium to that in the sacral body on both sides, and additional 2.0 mm screws were inserted in the sacroiliac joint to prevent counter rotation.

In the TK, a 1.4-mm K-wire was inserted from the lateral side of the ilium wing to the sacral body, penetrating out to the lateral side of the opposite ilium wing. The tip of the K-wire passing out from the ilium body was bent, pulled back, bent again, and cut.

**Figure-1:**
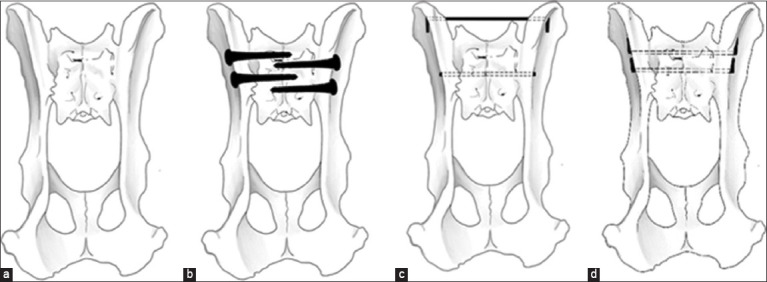
Various fixation methods of the bilateral luxation in cat’s pelvic models. (a) Control, (b) double lag screws, (c) one transilial pinning with one trans-iliosacral pinning, and (d) double K-wire trans-iliosacral pinning technique.

In the DK, double K-wires were inserted from the ilium wing through the sacrum body, emerging from the opposite ilium wing. Both tips of the K-wire were bent, pulled back, bent again, and the remaining K-wire was cut. The fixation methods used in this study are shown in Figure-1.

### Mechanical testing

**Figure-2:**
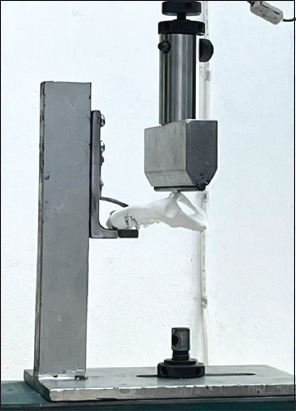
Mechanical loading on the ventral part of a pelvic bone model at the acetabulum level to simulate the physiological loading of the hindlimb in cats.

The feline pelvic models were secured with a mounting base at the sacral body in an upside-down position to stabilize the model. A compression force was applied from the top of the models to the pubic area, aligned with both acetabula, using a universal mechanical testing machine (Hounsfield Model H50KS, England) at a speed of 10 mm/min (Figure-2). Changes in distance were recorded in millimeters, and the force was recorded as Newton (N). The force was applied until the structure experienced fixation failure.

### Statistical analysis

Loads were recorded in Newton, and maximum bearing loads were calculated as averages and respective standard deviations. The data distribution was tested using the Shapiro test, while the Fligner-Killeen test was used to test variance. The maximum loads borne by various feline pelvic models were compared using a one-way analysis of variance. Significant differences between various groups were tested using Tukey’s Honest Significant Difference in the R programing language (R Core Team [2021]). R: A language and environment for statistical computing. R Foundation for Statistical Computing, Vienna, Austria. URL: https://www.r-project.org/.)

## Results

The group means of the maximum loads (measured in N) are presented as mean ± standard deviation, as shown in Table-1. A comparison of group means indicated that the DK group borne the highest maximum loads, while the control group had the lowest maximum bearing loads. The mean maximum load of the DK group was significantly different from that of the control group, whereas the maximum load of DK was not significantly different from that of the DS and TK groups.

**Table-1:** Maximum load (N) measured for each group and present as mean ± SD.

Group	Mean of the maximum load (N)	±SD
Control	91.84	±36.87
DS	137.90	±64.69
TK	120.18	±20.89
DK	183.86*	±30.86

* Indicates a significant difference (p < 0.05) between the given group and the control. DS=Double lag screws, TK=One transilial pinning with one trans-iliosacral pinning technique; DK=A double K-wire trans-iliosacral pinning technique.

**Figure-3:**
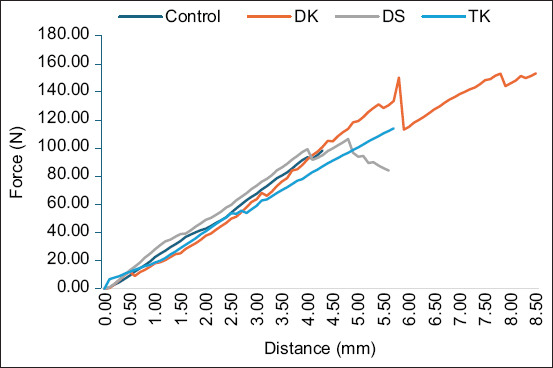
Average variation in the loads for each group during the mechanical testing.

Figure-3 shows a plot between the average load (n) and distance (mm) measured in the control, DS, TK, and DK groups. Although the slopes of the curves were similar, the peak maximum loads differed.

The stiffness (N/mm) of the samples measured for each group was 22.85, 22.20, 20.02, and 25.91 for the control, DS, TK, and DK groups, respectively. The stiffness of the DK group was the highest in this study, whereas the TK group had the lowest stiffness. This may be because the TK group uses only a pin that fixes the ilium with the sacral body, which acts as the pivot point, whereas the DS and DK groups have double fixation that eliminates the pivot point.

## Discussion

Bilateral sacroiliac joint luxation occurs in approximately 60% of the cases of total sacroiliac joint luxation in cats [24]. A pelvic canal narrowing exceeding 45% causes defecation problems and pain. Megacolon and constipation can result from pelvic narrowing [24]. Mismanagement post-pelvic fracture can jeopardize both patient survival and perceived quality of life.

Bilateral sacroiliac joint luxation in cats can be treated surgically or conservatively. To effectively treat surgically, DS should be used to attach the ilium wing to the sacral body [2, 6, 9]. Although effective, the double-screw technique encounters several challenges. Inserting screws into the limited space of the sacral body can be challenging, and the diameter of the screws must account for 25%–50% of the sacral wing in a normal cat [25]. 62% of the reported cases showed screw loosening following sacroiliac joint fixation as a result of pelvic narrowing [26]. The precision of sacral screw insertion was assessed using CT or fluoroscope [27, 28]. Small screws with a diameter under 2.0 mm necessitate advanced surgical methods for insertion. Our study indicates that the K-wire method for sacroiliac joint luxation treatment is less complicated than the double-screw method, involves fewer tools, and entails fewer steps. The quicker transiliosacral pinning method involves using K-wires, which, despite their speed advantage, risk soft tissue irritation due to their sharp tips [29]. To prevent soft tissue irritation and loosening of the implant, our study suggests bending the K-wire tip near the ilium cortex.

The DK group displayed the most bilateral sacroiliac joint luxation fixations, while the TK group showed the least. The TK procedure using the smaller space was simpler and less space-consuming compared to the DK technique, with minimal differences observed between TK, DS, and DK groups. In the clinical cases of bilateral sacroiliac joint luxation, the TK group may be useful for fixing the ilium wing to the sacral body, whereas the other K-wire, which fixes both ilium wings, can prevent pelvic narrowing post-fixation. The technique for transiliac pining in this study was based on the results of a study on the safe corridor for transiliac pin placement [30]. The average maximum load in the DS group was not significantly different from that in the TK and DK groups, suggesting that all techniques can be used for treating bilateral sacroiliac joint luxation. Furthermore, the TK and DK techniques require only two K-wires for fixation, making them cost-effective compared to the DS technique.

To measure the loading direction, we measured the physiological load of a cat’s weight bearing on the acetabulum. A previous study by Schnabl and Bockstahler [31] in 2015 reported that the reactionary force to the pelvic limb can be directed in three directions, with the ground reaction force being the most significant. The average peak vertical force on each hind limb of a cat (body weight between 3.8 and 6.6 kg) ranged from 19.92 to 20.02 N [32]. Based on these reports, all fixation methods in our study are suitable for treating sacroiliac joint luxation in cats.

To study the biomechanics of fixation devices, we used plastic PLA pelvic models printed using a 3D printer to eliminate the confounding factors present in dry or fresh bone samples [33]. The PLA’s similar density to normal cat tibia (1.3 g/cm^3^) and ulna (2.0 g/cm^3^) [34] avoided influences on mechanical outcomes when compared to tests on dry bone.

## Conclusion

A feline pelvic plastic model’s bilateral sacroiliac joint luxation was analyzed mechanically in this study. The DK group exhibited the greatest average maximum load capacity among all the tested groups. There was no significant difference in the maximum loads borne by all fixation groups. In cats, where there is limited space in the sacral body compared to dogs, K-wire fixation is a promising alternative to the widely used DS fixation for treating sacroiliac joint luxation. The transiliac pinning technique can help prevent complications related to sacroiliac joint luxation, like pelvic canal narrowing. In mechanical testing, the DK fixation technique outperforms, but the available sacral space for two K-wires is constricted, as is the case in the DS group. The TK technique uses less sacral space than the DK technique. The use of DK and TK techniques for sacroiliac joint luxation treatment in cats during surgeries can enhance surgical time efficiency and precision of device penetration. Future studies should investigate the clinical use of these fixation methods for sacroiliac joint luxation in cats.

## Authors’ Contributions

CT: Conceptualization, data analysis, and drafted the manuscript. TJ, KL, NK, PiP, and PaP: Conducted the experiment, data analysis, and drafted the manuscript. SP: Conducted the experiment and drafted and edited the manuscript. All authors have read, reviewed, and approved the final manuscript.
